# DNA looping by two 5-methylcytosine-binding proteins quantified using nanofluidic devices

**DOI:** 10.1186/s13072-020-00339-7

**Published:** 2020-03-16

**Authors:** Ming Liu, Saeid Movahed, Saroj Dangi, Hai Pan, Parminder Kaur, Stephanie M. Bilinovich, Edgar M. Faison, Gage O. Leighton, Hong Wang, David C. Williams, Robert Riehn

**Affiliations:** 1 Department of Physics, North Carolina State University, Raleigh, NC 27695-8202 USA; 2 Department of Pathology and Laboratory Medicine, University of North Carolina, Chapel Hill, NC 27599-7525 USA

**Keywords:** DNA methylation, MeCP2, MBD2, DNA compaction

## Abstract

**Background:**

MeCP2 and MBD2 are members of a family of proteins that possess a domain that selectively binds 5-methylcytosine in a CpG context. Members of the family interact with other proteins to modulate DNA packing. Stretching of DNA–protein complexes in nanofluidic channels with a cross-section of a few persistence lengths allows us to probe the degree of compaction by proteins.

**Results:**

We demonstrate DNA compaction by MeCP2 while MBD2 does not affect DNA configuration. By using atomic force microscopy (AFM), we determined that the mechanism for compaction by MeCP2 is the formation of bridges between distant DNA stretches and the formation of loops.

**Conclusions:**

Despite sharing a similar specific DNA-binding domain, the impact of full-length 5-methylcytosine-binding proteins can vary drastically between strong compaction of DNA and no discernable large-scale impact of protein binding. We demonstrate that ATTO 565-labeled MBD2 is a good candidate as a staining agent for epigenetic mapping.

## Introduction

Epigenetic regulation, the inheritable regulation of gene expression without changing the DNA sequence, often involves modifications of DNA bases and the specific binding of proteins to those modifications. One of the most widely studied epigenetic mechanisms is methylation of cytosine, which usually is simply referred to as DNA methylation. It is an inheritable chemical modification that regulates gene transcription [1]. The mechanism of this regulation is either by binding of dedicated proteins that serve as a reader of the epigenetic state [2], or by modulating the assembly of the transcription machinery directly [3]. Understanding the mechanisms through which DNA methylation influences gene expression is an active research field due to the growing evidence of its relation to human development and disease, and in particular aging [4], environmental response [5], and cancer [6].

Our focus is probing how members of the methyl-CpG-binding domain (MBD) protein family, which share a common methyl-binding domain [7], alter the conformation of DNA molecules. Our interest arises from two lines of thought. Firstly, demonstrating that an MBD protein compacts DNA as part of binding leads to the suggestion that the mechanism of modulation of transcriptional activity can be an effect of a steric compaction. Such mechanisms have been explicitly proposed for some MBD [8]. Secondly, MBD binding has been established as a label in epigenetic mapping technologies, and the lack of compaction would be a necessary condition for a specific epigenetic label. Such mapping technologies are emerging in a niche where bisulfite-based sequencing has remained very difficult, such as for single cells [9–11], or where the variability over large scales (>100 kbp) is required without single-base resolution but with high throughput.

Mapping of methylation patterns on extended DNA molecules intrinsically yields single-molecule information, and there are three main pathways to obtaining CpG methylation patterns using fluorescent probes [12]. The first is using antibody probes, which however works best in the context of fixed and denatured DNA [13, 14]. Thus this approach is not compatible with high-throughput DNA stretching in nanochannels that we employ. The second approach is covalent labeling of short motifs using methylation-specific proteins or methylases, to create a barcode-like pattern on DNA molecules [15]. Usually the flanking sequence of the CG site will strongly influence the specificity. The third method is the use of DNA-binding proteins to investigate the epigenetic information [16, 17]. In this paper, we expand on that last method by investigating two full-length MBD proteins that could be used provide methylation-specific labeling of DNA molecules.

All MBD proteins have a methyl-CpG-binding domain that enables them to specifically bind to methylated CpG sites. We focus on the family of proteins related to MeCP2. Members of this family with methylation-specific binding are MeCP2, MBD1, MBD2, and MBD4 [18]. Based on their specificity in binding to methylated CpG substrates, MBD proteins are good candidates for mapping methylation patterns on stretched DNA [16, 17]. Previous publications demonstrating mapping using MBDs have used either capillary force DNA stretching combined with a peptide from MBD1 as a probe [16], or nanofluidic channel stretching combined with an MBD peptide from MeCP2 [17]. While both studies demonstrated specific labeling, and both utilized only the methyl-binding domain from the respective protein, Lim et al. [17] reported shortening of the DNA while Cerf et al. [16] did not report this phenomenon.

The stretching technique employed by Lim et al. is confinement to nanofluidic channels with a cross-section of $100\,\hbox {nm}\,\times 100\,\hbox {nm}$ to $200\,\hbox {nm}\,\times \,200\,\hbox {nm}$, which extends DNA through interplay of excluded volume (self-avoidance), DNA stiffness, and confinement [19]. DNA is in an equilibrium state, and thus free to fluctuate and change its conformation as proteins bind, buffer conditions change, or proteins perform catalytic reactions [20]. Studies of DNA configurations in nanochannels under protein exposure have yielded insights into the modification of the mechanical parameters of DNA. Of specific interest for this paper is the probing of DNA–protein–DNA bridges, which can stabilize DNA loops or manifest themselves in an apparent modified effective DNA width. The formation of such DNA–protein–DNA bridges scales with the rate of DNA–DNA contacts, and thus is strongly dependent on DNA concentration. Typical concentrations of in vitro assays (10s of $\upmu \hbox {g}/\hbox {ml}$) are far too low to mimic the environment of a cell nucleus. The high local concentration of nanochannel-stretched DNA ($>100\,\upmu /\hbox {ml}$), while still not quite at a physiological level, leads to a much higher rate of DNA–DNA contacts [21]. In addition, nanochannel stretching requires no tethering and lacks external stresses, thus making nanochannel stretching an excellent platform to test protein-mediated DNA–DNA interactions and DNA–protein–DNA bridge formation.

In this study, we focus on two proteins from the MBD family, MBD2 and MeCP2. MBD2 interacts with the nucleosome remodeling and histone deacetylation (NuRD) complex [22]. It is expressed throughout the body [23]. Studies have shown that MBD2 helps NuRD target methylated CpG sites to modify the chromatin and regulate gene transcription [24, 25]. We are not aware of a co-operative mechanism in MBD2 binding, including in vivo correlations with the density of target sites [26]. MeCP2 is highly expressed in the brain and well-studied because of its impact on brain function and disease [27]. MeCP2 is capable of co-operative binding to DNA oligomers that contain a single specific binding site [28], with salt-dependent binding constants and cooperativity. However, this cooperativity in general decreases the specificity of binding. Interestingly, an effect of methyl CpG density-dependent binding, with higher affinity for higher densities, is apparent in in vivo data [26]. That points to a second possible mechanism of cooperativity that is regulated through the DNA density itself. In particular, MeCP2 has been shown to be able to bridge and loop DNA molecules [8, 29]. While the apparent compaction of DNA by an MBD peptide from MeCP2 [17] suggests the presence of MBD dimers that are able to form protein bridges between DNA molecules, surprisingly no cooperativity was observed in the formation of dimers on short substrates [30]. That may point to the fact that the compaction observed in [17] is driven by the fluorescent label.

Here we show that while both proteins bind with similar affinity to their target sequences, the extension of DNA is very different for both cases. In particular, MBD2 did not change the conformation of DNA molecules, and the extension is insensitive to fluorescent labeling of the MBD2. In contrast, MeCP2 compacted the DNA molecules to less than 50% of their native extension. By using atomic force microscopy (AFM), we show that the probable mechanism for compaction by MeCP2 is the formation of DNA–protein–DNA bridges that create local loop configurations. We thus believe that MBD2 is a superior epigenetic probe to MeCP2, and that full-length proteins can not only be used for labeling reagents, but may be performing better than the isolated MBD peptides that have been used in the past.

## Materials and methods

### Nanochannel fabrication

Devices with nanochannels and microchannels were fabricated on fused silica wafers. Nanochannels ($180\,\hbox {nm}\,\times \,180\,\hbox {nm}$) were patterned by electron beam lithography and microchannels were patterned by optical lithography. We obtained both nanochannels and microchannels by reactive ion etching and sealed the device with a second fused silica substrate by thermal bonding [31].

To prevent proteins from sticking to the surface, we used dopamine-mPEG to passivate the interior surfaces of the device [32]. The device was first exposed to ${2}\,\hbox {mg}/\hbox {ml}$ of dopamine in ${10}\,\hbox {mM}$ Tris buffer (pH 8.0) for 2 h and then coated using ${100}\,\hbox {mg}/\hbox {ml}$ of methoxypolyethylene glycol amine (mPEG-$\hbox {NH}_2$, MW=5000 kDa) in ${10}\,\hbox {mM}$ Tris buffer (pH 8.0) overnight.

### Biological materials

**Fig. 1:**
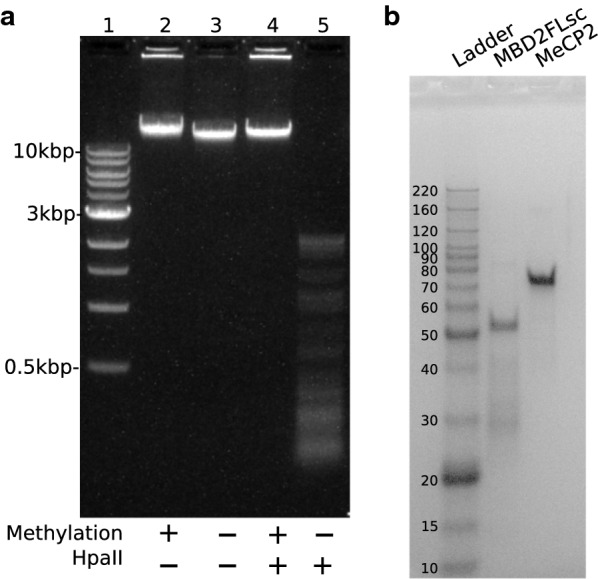
**a** Agarose gel electrophoresis for verification of cytosine methylation. Lane 1 is a DNA ladder, lane 2 is methylated $\lambda$-DNA, and lane 3 is unmethylated $\lambda$-DNA. Methylated $\lambda$-DNA was not digested by HpaII (lane 4), while the unmethylated $\lambda$-DNA was digested by HpaII (lane 5). **b** SDS-PAGE demonstrating purity of proteins used in experiments

Methylcytosine-free linearized $\lambda$-DNA (48.5 kbp) was purchased from Sigma-Aldrich. We used M.SssI (New England Biolabs) to methylate $\lambda$-DNA following the supplier protocol. CpG methylation was confirmed by showing the suppression of digestion by HpaII (New England Biolabs) after methylation, as presented in Fig. 1a. The methylase was finally removed by purification using a QIAEX II Gel Extraction Kit (Qiagen). In figures throughout this manuscript, DNA without 5-methylcytosine is labeled as “C-DNA”, and DNA carrying the maximum density of 5-methylcytosine is labeled as “5mC-DNA”.

MBD2FLsc is a full-length human MBD2 single-chain construct comprising MBD2 (amino acids 150–393) and the scMBD2-GATAD2A expressed in *E. coli* and purified by nickel affinity chromatography [24]. The as-purified MBD2FLsc is labeled as “MBD2” in figures. For part of the experiments MBD2FLsc was labeled using ATTO 565-maleimide (ATTO-TEC), and purified by gel filtration chromatography (Sephadex G-50, GE Healthcare). The labeled MBD2FLsc is labeled “ATTO-MBD2” in figures. The recombinant human MeCP2 protein produced by mammalian expression system (Accession P51608) with a 6His tag at the C-terminus was purchased from Novoprotein. The purity of both proteins was confirmed using SDS-PAGE (Fig. 1b).

To test the binding affinity of proteins before and after labeling, we performed fluorescence anisotropy measurements using a FAM-labeled dsDNA with one methylated CpG binding site as the substrate [24, 33]. We performed protein titrations with $${24}\,\hbox {nM}$$ of the DNA substrate in a working buffer (HEPES $${20}\,\hbox {mM}$$, NaCl $${50}\,\hbox {mM}$$, $$\hbox {MgCl}_2$$$${3}\,\hbox {mM}$$, EDTA $${3}\,\hbox {mM}$$, pH 7.5). The fluorescence anisotropy was measured using a PTI QuantaMaster 40 (Horiba), and the results were fitted using the Langmuir (single-binding site) model.

### Fluorescence microscopy

Both methylated and unmethylated $\lambda$-DNA were stained using YOYO-1 (Life Sciences) at a 10:1 molar ratio of base pairs to dye before imaging. To prevent non-specific binding of the protein, we added mPEG-$\hbox {NH}_2$ to the working buffer with final concentration at ${30}\,\hbox {mg}/\hbox {ml}$. For the MeCP2 experiment, we further added 1$\%$ of polyvinylpyrrolidone (PVP).

We first incubated the $\lambda$-DNA with the respective protein in our working buffer for 30 min, if applicable. The solution was then introduced into the device using pressurized nitrogen, and DNA was driven into nanochannels using hydrostatic pressure. When a molecule entered, the pressure was removed which caused molecules to stop in the nanochannel. Imaging was performed on an inverted fluorescence microscope (Nikon TE-2000) with a Nikon 60$\times$ oil immersion objective (*NA*=1.40) in near TIRF mode, and the data were collected by an emCCD (Andor iXon Life) and a QV2 image splitter (Photometrics). For observation of YOYO-1-stained DNA, we used a ${488}\,\hbox {nm}$ laser for illumination and a 525/40 bandpass filter for collection. For the protein signal (ATTO 565), we used a ${561}\,\hbox {nm}$ laser and a 600/35 bandpass filter. The data were analyzed using ImageJ and Matlab (Mathworks).

Bare nanochannel-stretched DNA shows a constant ratio between contour length and extension and thus a constant brightness along the molecule [19]. We have found that this assumption does not hold for DNA strongly compacted by a protein, and thus needed to find different way to characterize the size of a DNA configuration. For each data frame we calculate the autocorrelation function of the signal intensity *I*(*x*) along a DNA molecule

1 $$\begin{aligned} {\mathcal {C}}(\delta x)=\frac{\sum _i I(x_i+\delta x)I(x_i)}{\ell -\delta x}\,\,, \end{aligned}$$

where *I* is the background-corrected intensity, $x_i$ are the positions of the individual pixels, $\ell$ is the length of the analysis window, and $\delta x$ are displacements that are integer multiples of the pixel size. The autocorrelation function can be averaged over all frames to form $\left<{\mathcal {C}}\right>(\delta x)$. For a molecule with constant intensity along the channel, the autocorrelation function would be a triangle with a full width at half maximum that matches the extension of the molecule. However, we explicitly do not want to make that assumption and instead obtain the length information by calculating the radius of gyration of the autocorrelation function

2 $$\begin{aligned} R_g=\sqrt{\frac{\sum \limits _{\delta x}\left<{\mathcal {C}}\right>(\delta x)\cdot (\delta x)^2}{\sum \limits _{\delta x}\left<{\mathcal {C}}\right>(\delta x)}}\,\, \end{aligned}$$

where sums run such that $\delta x$ sweeps $-\ell /2 ... \ell /2$, which is chosen about 3 times the average extension of observed molecules. For a uniformly stretched molecule that has an intensity profile that is a Gaussian-widened boxcar function of length *L* [31], we obtain $R_g=\sqrt{\frac{L^2}{6}+\sigma ^2}$. Here $\sigma ^2$ is the sum of the variance of the point spread function of the microscope objective, and the variance due to the blurring by thermal fluctuations of the system. For large extended molecules, $R_g$ scales linearly with the extension along the channel axis, while for compacted molecules the $\sigma$-term begins to dominate.

The mean extensions for a given condition was found by first determining the $R_g$ for each molecule of the set separately, and then finding the numerical mean and variance of the distribution. From these, the error of the mean was determined and a Gaussian distribution function was determined that is used in the histogram plots of $R_g$. The Gaussian assumption is expected to hold well for large molecules [34].

The advantage of this approach is that no assumption about the underlying fluorescence profile along the DNA molecule has to be made, and that averaging of correlations functions is robust because no fitting of any kind is required prior to averaging. Importantly, the more compacted the molecule is, the smaller the $R_g$ value will be. By comparing the $R_g$ result with and without incubation with a protein, we can determine whether a protein can compact the DNA molecule without requiring a detailed understanding of the stretching process.

### Atomic force microscopy

For atomic force microscopy (AFM), we used a 7,163-bp linear DNA substrate which contains a 1,697-bp methylated CpG-rich region that is flanked by 2,742-bp and 2,724-bp CpG-free regions [25]. For MeCP2, the DNA substrate and the protein were diluted in AFM imaging buffer (HEPES 20 mM, Mg(OAc)$$_2$$$${10}\,\hbox {mM}$$, NaCl $${100}\,\hbox {mM}$$, pH 7.5), mixed together and deposited on freshly peeled mica. For MBD2FLsc, we first mixed the protein and DNA and then diluted the sample in AFM buffer before deposition. The final MeCP2 concentration deposited on mica was $${7.5}\,\hbox {nM}$$, and the MBD2FLsc concentration was $${14}\,\hbox {nM}$$. The mica samples were then washed with filtered deionized water and dried with nitrogen. We used a MFP-3D-Bio AFM from Asylum Research with Pointprobe®PPP-FMR probes (Nanosensors, $$\approx$$$${2.8}\,\hbox {N}/\hbox {m}$$) to image the sample at a scan resolution of $${5.9}\,\hbox {nm}$$ and a scan rate of $${3}\,\upmu \hbox {m}/\hbox {s}$$. The data were analyzed using Asylum Research software and Matlab.

We introduce two quantitative measures for DNA configuration. First, we want to determine whether an object in the AFM image is indeed a single DNA molecule, or whether it is actually a complex of two or more molecules. To this end, we count the number of DNA ends in an image. A single molecule should have two ends at most. Note that one or two ends are possible if the molecule is randomly deposited in a configuration that includes a loop. We can further introduce a quantitative measure for the “loop count” within the single molecules by counting the number of unoccupied areas that are full enclosed by DNA contour within a molecule. This numerical measure is a 2-d projection of the 3-d configuration of the molecule prior to deposition, and thus an over-count of the physical number of loops that exist in free solution. However, it is operationally very robust, while determining the number of physical loops that are stabilized by bound protein is considerably slower and carries a higher uncertainty. Both quantitative measures are illustrated in Fig. 6, and the definition of the “loop count” is specifically illustrated in Fig. 6c.

## Results

**Fig. 2:**
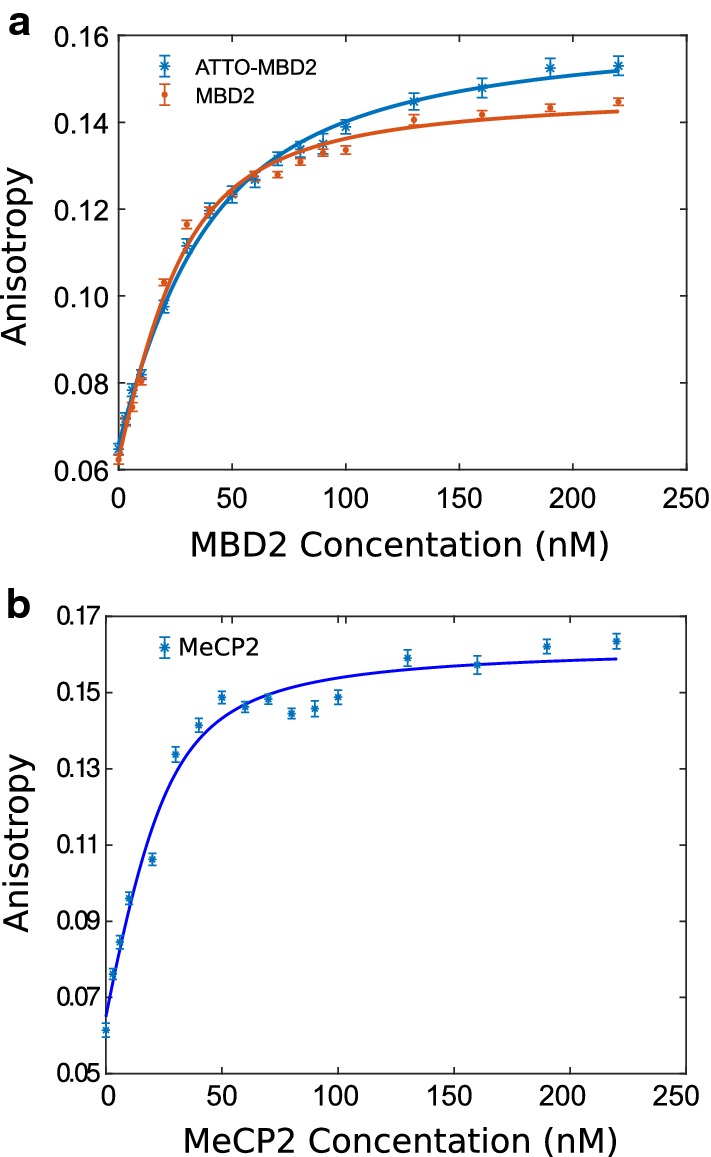
Fluorescence anisotropy titration of protein binding to ${24}\,\hbox {nM}$ methylated dsDNA substrate. **a** Labeled (blue *) and unlabeled MBD2FLsc (red •) yielded affinities of $24.5\,\pm 4.6\,\hbox {nM}$ and $11.9\,\pm 3.8\,\hbox {nM}$, respectively. **b** MeCP2 yielded an affinity of $7.5\,\pm \,4.3\,\hbox {nM}$. Error bars are standard deviations over 3 measurements

Protein modification can lead to a modulation of binding affinities, both between the protein and its substrate as well as between proteins. This is particularly important since we are interested in finding an MBD protein for potential use as a epigenetic labeling agent. The maleimide chemistry underlying the labeling of MBD2FLsc is prone to disrupt protein configuration. In particular, attack of protein disulfide bonds may cause the structure of MBD2FLsc to change, thus lowering its binding affinity to DNA. Fluorescence anisotropy titration yielded a $K_d$ of $25 \pm 5\, \hbox {nM}$ and $12\pm 4\,\hbox {nM}$ for labeled and unlabeled MBD2FLsc, respectively (Fig. 2a). The value for unlabeled MBD2FLsc is consistent with [24], and we conclude that a large fraction of the labeled MBD2FLsc population retained its ability to bind to methylated CG sequences.

**Fig. 3:**
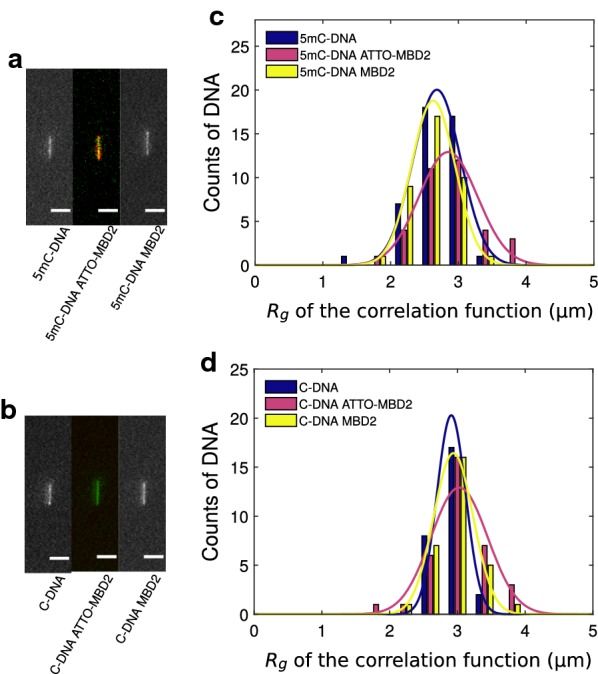
Nanochannel stretching of YOYO-1 stained DNA under MBD2FLsc binding. **a** Fluorescence images of methylated DNA (left), methylated DNA with labeled MBD2FLsc (center, ATTO 565-labeled MBD2FLsc in red and methylated $\lambda$-DNA in green), and methylated DNA with unlabeled MBD2FLsc (right). **b** Fluorescence images of unmethylated DNA (left), unmethylated DNA with labeled MBD2FLsc (center with ATTO 565 labeled MBD2FLsc in red and methylated $\lambda$-DNA in green), and unmethylated DNA with unlabeled MBD2FLsc (right). The scale bar represents ${5}\,\upmu \hbox {m}$. **c** Histograms of $R_g$ for methylated DNA (blue: bare DNA, *N*=44, magenta: ATTO 565-MBD2FLsc, *N*=35, yellow: MBDFLsc, *N*=38). **d** Histograms of $R_g$ for unmethylated DNA (blue: bare DNA, *N*=27, magenta: ATTO 565-MBD2FLsc, *N*=34, yellow: MBDFLsc, *N*=30)

Figure 3a, b shows representative images of single nanochannel-stretched DNA molecules that were exposed to MBD2FLsc with and without DNA substrate methylation, respectively. Binding of labeled MBD2FLsc to unmethylated DNA was observed at a greatly reduced density when compared to methylated DNA (Fig. 3b). The magnitude of that reduction and the specificity arising from it is treated in detail at a later point of the manuscript. On first inspection, MBD2FLsc does not strongly change the extension of either methylated or unmethylated DNA, either with or without fluorescent labeling of the MBD2FLsc. This impression confirmed by the quantitative analysis (Fig. 3c, d), with numerical results presented in Table 1.

**Table 1:** Summary of $R_g$ for nanochannel-stretched DNA with MBD2FLsc as determined in Fig. 3

Substrate	Bare DNA (μm)	ATTO-MBD2 (μm)	MBD2 (μm)
Methylated DNA	$2.68\,\pm \,0.5$	$2.85\,\pm \,0.07$	$2.63\,\pm \,0.05$
Unmethylated DNA	$2.91\,\pm \,0.04$	$3.03\,\pm \,0.07$	$2.94\,\pm \,0.05$

The error levels are the standard error of the mean

We observed a significant difference between the extension of methylated and unmethylated DNA, with a larger extension for unmethylated DNA (*p* < 0.015). That is contrary to the finding by Sun et al. who find that methylated DNA is more extended [35]. However, it is consistent with the decreased persistence length of DNA sequences containing of 5-methylcytosine reported by Ngo et al. [36]. Another possibility for a decreased extension of methylated DNA compared to unmethylated DNA is a drop in DNA persistence length due to damage during the methylation and purification protocol, which is exacerbated by the relatively large length of our substrate. Note that $R_g$ values for DNA in Fig. 3 are consistent with the observed extension of about $6\,\upmu \hbox {m}$ according to the argument in the "Materials and methods" section.

The $R_g$ variations between different protein conditions for unmethylated DNA were not statistically relevant (all likelihoods of null-hypothesis* p* >0.24). Some variations are expected in light of the strong dependence of the extension with nanochannel width [21], our manufacturing precision over a 4-in. wafer, and the variability of the thickness of the channel coating that we apply. For methylated DNA substrates, the $R_g$ for bare DNA and unlabeled MBD2FLsc were not significantly different (*p* < 0.43). However, fluorescently labeled MBD2FLsc lead to an approximately 4% larger $R_g$ (*p*< 0.05), possibly due to a larger effective volume of the labeled protein. Importantly, the effects of smaller extension of methylated DNA substrates (compared to unmethylated) and larger extension of DNA with bound ATTO 565-labeled MBD2FLsc (compared to bare methylated substrates) cancel each other out, so that the difference in extension between bare unmethylated DNA and methylated DNA with bound labeled MBD2FLsc is statistically insignificant (*p* < 0.52).

The characterization up to this point was performed at a ratio of one MBD2FLsc per target site along DNA. We next characterized whether this result is sensitive to the concentration of ATTO 565-MBD2FLsc. We summarize these results in Table 2. We find only a weak dependence of $R_g$ for methylated substrates as a function protein concentration when the ratio [CpG]:[MBD2] is varied from 1:0.5 to 1:1.7, with the lowest likelihood of the zero-hypothesis being* p*=0.38. For unmethylated substrates, the dependence on protein concentration is stronger, with a mild contraction at [CpG]:[MBD2]=1:1.7. However, the level of significance within the statistics of our experiments is low, with* p* < 0.26 for hypothesis that the $R_g$ is independent of protein concentration.

**Table 2:** Summary of $R_g$ for nanochannel-stretched DNA with ATTO-565-labeled MBD2FLsc as a function of concentration of MBD2FLsc determined from the dataset underlying Fig. 4

Substrate	1:0.5 (μm)	1:1 (μm)	1:1.7 (μm)
Methylated DNA	$2.73\,\pm \,0.10$	$2.85\,\pm \,0.07$	$2.72\,\pm \,0.11$
Unmethylated DNA	$3.03\,\pm \,0.06$	$3.03\,\pm \,0.07$	$2.85\,\pm \,0.08$

Columns are labeled by the ratio [CpG]:[MBD2]

The error levels are the standard error of the mean

**Fig. 4:**
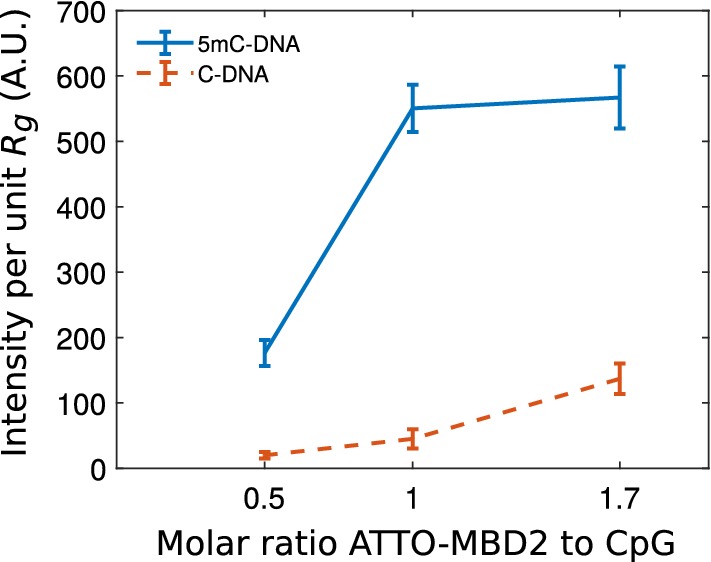
Protein fluorescence signal intensity per unit $R_g$ of nanochannel-stretched DNA as function ATTO 565-labeled MBD2FLsc concentration. Blue (upper) curve is for methylated substrates and red (lower) curve is for unmethylated substrates. Error bars represent standard deviations

The same dataset lets us determine the protein concentration that leads to the highest specificity, which we define as the ratio of bound fluorescent MBD2FLsc bound to a methylated and an unmethylated $\lambda$-DNA substrate, respectively. For densely bound proteins in fluorescence imaging, the number of proteins is proportional to the integrated intensity of the fluorescence signal. To account for a possible variation in DNA lengths due to handling, the fluorescence signal was normalized to $R_g$, which is proportional to the DNA extension in a nanochannel. As anticipated, the specificity is a function of the concentration of the labeling agent since the number of proteins is limited to the number of recognition sites (Fig. 4). We observe that the number of bound proteins to the methylated substrate plateaus at parity between protein concentration and concentration of CpG sites. The non-specific binding to the unmethylated substrate does not show a plateau. At the point of parity between MBD2FLsc and binding site concentrations, the specificity reaches approximately a factor of 10.

**Fig. 5:**
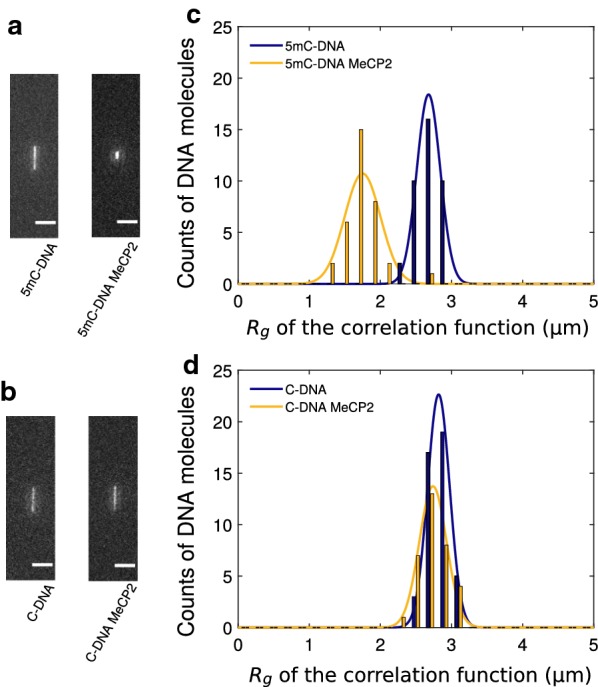
Nanochannel stretching of YOYO-1 stained DNA under MeCP2 binding. **a** Fluorescence images of methylated $\lambda$-DNA (left) and methylated DNA with MeCP2 (right). **b** Fluorescence images of unmethylated $\lambda$-DNA (left) and unmethylated DNA with MeCP2 (right). The scale bar represents ${5}\,\upmu \hbox {m}$. **c** Impact of MeCP2 binding on $R_g$ for methylated DNA (blue bare DNA* N*=38, yellow with MeCP2* N*=34). **d** Impact of MeCP2 binding on $R_g$ for unmethylated DNA (blue bare DNA* N*=44, yellow with MeCP2* N*=33)

Turning to MeCP2, we first confirmed that the MeCP2 binding affinity is appropriate for the protein using fluorescence anisotropy (Fig. 2b). We find a $K_d$ of $7.5\,\pm 4.4\,\hbox {nM}$, which is in agreement with [28]. For the imaging of MeCP2 on DNA confined to nanochannels, we added 1% of polyvinylpyrrolidone (PVP) inside the working buffer to improve surface passivation [17]. Fluorescence images of methylated and unmethylated $\lambda$-DNA under nanochannel stretching with MeCP2 show strong compaction of methylated substrates (Fig. 5). Quantitative analysis shows that the $R_g$ of unmethylated DNA contracted only very slightly upon exposure with MeCP2 (Table 3), with* p* < 0.09. That is in contrast to methylated DNA, which contracted strongly upon MeCP2 exposure ($\hbox {p}<6 \times {10}^{-7}$). We did not attempt to label MeCP2 since the compaction of DNA both is evidence of DNA binding, as well as an undesired feature for any epigenetic profiling application. A similar difference between methylated and unmethylated DNA was observed as in the first data set, with a shorter extension for methylated DNA (*p*< 0.0015).

**Table 3:** Summary of $R_g$ for nanochannel-stretched DNA as determined in Fig. 5 for MeCP2

Substrate	Bare DNA (μm)	MeCP2 (μm)
Methylated DNA	$2.68\,\pm \,0.03$	$1.75\,\pm \,0.04$
Unmethylated DNA	$2.82\,\pm \,0.02$	$2.74\,\pm \,0.03$

The error levels are the standard error of the mean

**Fig. 6:**
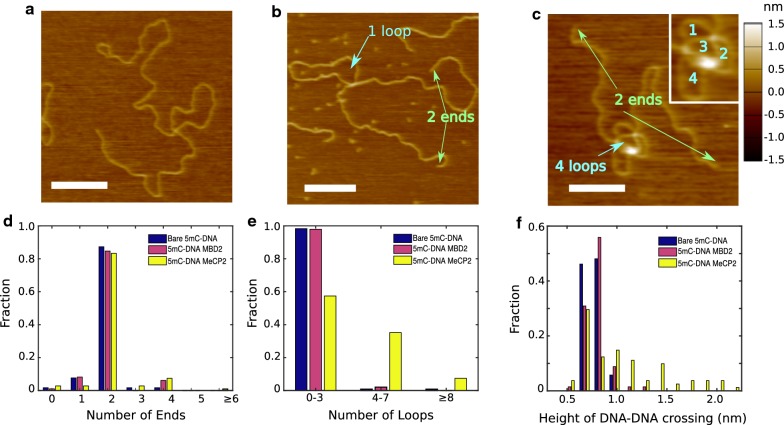
Atomic force microscopy (AFM) of methylated substrates under various conditions. AFM of bare methylated dsDNA oligomer **a**, the same oligomer with MBD2Flsc **b**, and with MeCP2 **c**. Scale bars are ${200}\,\hbox {nm}$. The green arrows point at ends, and cyan arrows point at loops. The inset in **d** illustrates the counting method for loops. The distribution of number of free ends (**d**) and the distribution of number of loops **(e)** for DNA or DNA–protein complexes was determined from such images (bare DNA* N*=118, MBD2FLsc* N*=98, MeCP2* N*=108). **f** Height of isolated DNA–DNA crossings (bare DNA* N*=52, MBD2FLsc* N*=68, MeCP2* N*=83)

We consider four mechanisms for the compaction of DNA by MeCP2 [20]. First, the compaction could be due to molecular crowding as reported by Zhang et al. [37], but note that this is unlikely in light of our protein concentration. Furthermore, if crowding were the cause, then a similar compaction would have been expected for methylated and unmethylated substrates. The second possibility is a condensation that effectively lowers the contour length through packing into a chromatin-like filament [38, 39]. The other two mechanisms could be a shortening through a local modulation of physical parameter such as the persistence length and the effective width (the inverse to [40]), or the formation of localized loops that are stabilized by MeCP2 locking the looped configurations that form as part of thermal fluctuations. To distinguish between the latter three mechanisms, we used atomic force microscopy (AFM) to image the conformations of DNA molecules. In order to obtain configurations that are easy to analyze, we chose a linear DNA as the substrate that carries methylated CpG-rich and CpG-poor regions [25]. Example images with bare DNA, MBD2FLsc, and MeCP2 are shown in Fig. 6a–c. In general, DNA appeared more compact in presence of MeCP2 than MBD2FLsc, with an apparent excess of looped configurations for MeCP2, suggesting the compaction by that protein in nanochannels is driven by the formation of loops. We also observed sporadic large DNA–protein clusters formed in presence of MeCP2 at high concentrations.

In the "Materials and methods" section, we describe a pathway for quantifying DNA configuration based on ends and open loops. For bare DNA, in excess of 90% of molecules were non-overlapping as indicated by the fraction of DNA with zero, one, and two ends (Fig. 6d). That number decreased marginally for MBD2 (*p*< 0.53) and somewhat for MeCP2 (*p*< 0.04), but in all cases more 80% of molecules were isolated. Moving to the count of visible DNA loops for molecules with two or less ends (Fig. 6e), we find that MBD2FLsc does not alter the conformation of DNA molecules (*p*< 0.53). 98.3±1.2% of bare DNA and 98.3±1.2% of DNA with MBD2FLsc had between 0 and 3 loops. That value dropped to 57.4±4.2% when MeCP2 was added to DNA, which is a significant effect ($\text{p} < 7\,\times \,10^{-14}$). At the same time, 35.2±4.3% of molecules showed 4 to 7 loops, and 7.4±2.4% showed 8 or more loops.

To determine whether the loops are linked to the presence of protein, we determined the apparent height of DNA–DNA cross-overs for all three conditions (Fig. 6f). The height of both bare DNA and DNA with MBD2FLsc follow an approximately monomodal distribution with mean height and standard deviation of $0.76 \,\pm \, 0.08\,\hbox {nm}$ and $0.80\,\pm \,0.12\,\hbox {nm}$, respectively. The difference is at the border of statistical significance (*p*< 0.1). We cannot resolve the exact location of proteins along the DNA, and thus cannot determine whether the small increase is due to protein that is bound in vicinity of the cross-over on one segment, or to both DNA at the cross-over point.

The distribution of heights at DNA cross-overs in presence of MeCP2 does not follow a monomodal distribution, and is rather characterized by the location of apparent protein clusters of varying size at DNA–DNA contact points with a mean cross-over height of $1.12\,\pm \,0.61\,\hbox {nm}$. Difference to both DNA and MBD2FLsc is statistically significant with $\hbox {p}< 8\,\times \,10^{-4}$. Interestingly, a subset of cross-overs with MeCP2 displays the same height as for bare DNA, likely due to the fact that these were not true loop anchoring points before deposition, but rather are random cross-overs caused by deposition onto a 2-dimensional substrate. We further note that in dense configurations the apparent height could point to a collection of more than two co-localized DNA segments in the probed volume of the AFM tip.

## Discussion

We have demonstrated that ATTO 565-labeled MBD2FLsc is a promising candidate for epigenetic mapping applications, since unmethylated DNA without labels and methylated DNA with labels exhibit the same extension. We have further demonstrated a specificity of about tenfold on our substrate. Note that the apparent specificity is likely a function of the substrate. Specifically, $\lambda$-DNA carries only about 300 CpG site in 48 kbp overall. That means that non-specific sites are considerably more abundant than specific sites. Furthermore, footprint-limited binding is well documented for DNA-binding proteins with multiple binding sites, such that the specific binding sites are less than fully occupied [41]. This can be reconciled with our finding of maximum occupancy close to equal concentrations of MBD2 and substrate (Fig. 4) by noting that not all protein may be active.

The compaction effect for MeCP2 agrees with prior studies [8, 29], but a number of concluding remarks are warranted. Importantly, the compaction described here does not require interaction with any additional proteins, such as nucleosomes, ribosomes, or similar. We also note the compaction reported by us is the result of DNA methylation. Within the scope of this paper, we are not able to resolve whether the DNA–protein–DNA bridges contain single proteins or are in actual fact DNA–protein–protein–DNA bridges. However, we can make the statement that both scenarios lead to a co-operative effect that will enhance the effective binding of MeCP2 to highly methylated sequences.

This can be seen from a thermodynamic argument: Since the closing of a loop requires two helices of DNA, the binding probability of a protein must scale with the square of the DNA concentration. If a randomly formed DNA–DNA contact is stabilized by one or two copies of MeCP2, the recruitment of further MeCP2 becomes thermodynamically more favorable. As more and more protein are recruited, the DNA configuration will become increasing denser, thus further enabling the binding of MeCP2. While interaction of MeCP2 with other nucleoproteins certainly is required for full function, we suggest that the repression of expression of methylated substrates by MeCP2 can partially explained by this compaction. This is in direct contrast to MBD2FLsc, which does not impact the DNA configuration. Instead, MBD2 requires other proteins of the NuRD complex for regulation of gene expression [24].

## Conclusions

In conclusion, ATTO 565-labeled MBD2FLsc appears to be a promising labeling agent specificity targeting methylated CpG sites without changing DNA length under nanochannel stretching. We have found no evidence that MBD2FLsc binding is modulated by binding of proximal MBD2FLsc. On the other hand, MeCP2 shows a strong impact on the organization of DNA and is unlikely to be a good labeling agent.

## Data Availability

The datasets during and/or analyzed during the current study available from the corresponding author on reasonable request.
